# Comparação entre cirurgia aberta e endovascular no tratamento do aneurisma da artéria poplítea: uma revisão

**DOI:** 10.1590/1677-5449.008817

**Published:** 2018

**Authors:** Ana Fernanda Fagundes Gonçalves, Carlos Augusto Pelek, Lorena Slusarz Nogueira, Renan Francisco de Carvalho, Matheo Augusto Morandi Stumpf, Ricardo Zanetti Gomes, Ana Claudia Garabeli Cavalli Kluthcovsky

**Affiliations:** 1 Universidade Estadual de Ponta Grossa – UEPG, Faculdade de Medicina, Ponta Grossa, PR, Brasil.

**Keywords:** aneurisma, artéria poplítea, procedimentos endovasculares

## Abstract

Os aneurismas de artéria poplítea correspondem a 70% dos aneurismas periféricos e o tratamento é cirúrgico, com controvérsias sobre os resultados da via endovascular. Este estudo objetivou realizar uma revisão da literatura sobre a comparação entre cirurgia aberta e endovascular no tratamento dos aneurismas da artéria poplítea. A pesquisa foi realizada utilizando os termos apropriados nos portais de periódicos LILACS e MEDLINE, com a seleção de 15 artigos. Um total de 5.166 procedimentos cirúrgicos foram comparados, sendo 3.930 cirurgias abertas e 1.236 cirurgias endovasculares. A cirurgia aberta com *bypass* venoso continua sendo o padrão-ouro. A cirurgia endovascular apresenta menor tempo de internação e é uma opção viável em pacientes eletivos, com baixa expectativa de vida, alto risco cirúrgico, comorbidades e mais idosos, desde que tenham anatomia favorável para o procedimento. Contudo, são necessários estudos de longo prazo para estabelecer os reais benefícios e indicações das duas técnicas, como o ensaio clínico randomizado controlado.

## INTRODUÇÃO

 Os aneurismas da artéria poplítea (AAPs) são os mais frequentes entre os aneurismas periféricos, correspondendo a cerca de 70% dos casos. Acometem principalmente os idosos e cerca de 90% dos pacientes são do gênero masculino 1 . 

 Devido à posição anatômica profunda da artéria poplítea, a realização do exame clínico dessa região torna-se complicada, o que pode dificultar o diagnóstico dos AAPs, principalmente em casos assintomáticos. O diagnóstico desse tipo de aneurisma pode ser feito na ocorrência de trombose aguda do aneurisma (se apresentando como uma oclusão arterial aguda) ou através de embolizações distais, calcificações em arteriografias, palpação de massa pulsátil na região poplítea durante o exame físico 1 e métodos de imagem como ultrassom com Doppler, angiotomografia computadorizada ou angiorressonância e arteriografia, em alguns casos 1 ^-^ 3 . 

 Um aneurisma poplíteo possui recomendação cirúrgica para diâmetros acima de dois centímetros ou quando sintomático 4 . O tratamento cirúrgico pode ser feito através da cirurgia aberta, que é a abordagem mais utilizada, ou da cirurgia endovascular, método no qual se utiliza uma endoprótese 5 . 

 A cirurgia tradicional por via aberta consiste em uma ligadura proximal e distal ao aneurisma da artéria poplítea para exclusão desse segmento, com um *bypass* venoso contornando o aneurisma (embora um enxerto protético seja outra opção). Essa técnica pode ser simples quando não há necessidade de expor o aneurisma, isto é, quando não é necessária a secção de tendões. Entretanto, possui algumas desvantagens, pois as ligaduras proximal e distal podem deixar ramos geniculares que mantêm o aneurisma, permitindo desse modo o crescimento mesmo após a cirurgia. Isso pode causar síndrome compartimental de estruturas vizinhas, sendo necessária uma nova cirurgia. Uma técnica cirúrgica alternativa é a aneurismectomia seguida de interposição por um *bypass* protético entre os seguimentos proximal e distal normais da artéria poplítea. A abordagem tradicional, porém, é a mais utilizada 1 . 

 A técnica endovascular é menos invasiva, pois se implanta uma endoprótese pela região inguinal, e dessa forma ocorre a exclusão do saco de aneurisma da circulação 6 . Nesse procedimento, o aneurisma deve possuir um colo proximal e distal de no mínimo um centímetro de comprimento para que possa ser feita a fixação da endoprótese. Contudo, a artéria poplítea apresenta pontos de articulação pela flexão do joelho devido à sua posição anatômica. Desse modo, quando o aneurisma ultrapassa a linha articular, é necessária a implantação de uma endoprótese flexível que se adapte à anatomia local 7 . 

 Embora o tratamento dos AAPs possa ser realizado tanto por via aberta como por via endovascular 6 , ainda há controvérsias na literatura sobre as vantagens de uma técnica em relação à outra. Além disso, há poucos estudos nacionais e internacionais publicados comparando os resultados dessas duas abordagens cirúrgicas no tratamento dos AAPs. 

 Este trabalho objetivou realizar uma revisão da literatura com estudos que compararam a cirurgia aberta com a cirurgia endovascular no tratamento dos AAPs. 

## MATERIAL E MÉTODOS

 Trata-se de um estudo de revisão da literatura sobre a comparação da cirurgia aberta e a cirurgia endovascular no tratamento dos AAPs. Como fonte de material para a elaboração do artigo, foram utilizados os portais de periódicos da Literatura Latino-Americana e do Caribe em Ciências da Saúde (LILACS) e do Sistema *On-line* de Busca e Análise de Literatura Médica (MEDLINE), através da PubMed. 

 Para determinar quais os elementos fundamentais da questão de pesquisa e da construção da pergunta para a busca bibliográfica de evidências, aplicou-se a estratégica PICO 8 . As letras desse acrônimo representam paciente, intervenção, comparação e *outcomes* (desfechos), respectivamente. Nesta revisão, portanto, os descritores “aneurisma AND poplítea” foram utilizados para definir quais eram os pacientes a serem estudados; “cirurgia” foi o descritor utilizado para o critério de intervenção; e “endovascular” foi o descritor utilizado para a comparação à intervenção. Não foi utilizado descritor para o desfecho, pois os artigos analisados eram muito heterogêneos com relação aos objetivos (alguns avaliaram a patência primária no primeiro mês e outros anos após o tratamento, sendo que nem todos avaliaram tempo de internação hospitalar, por exemplo). 

 Houve delimitação do tempo de busca para os últimos 20 anos, pois o início do tratamento endovascular em AAP ocorreu nos anos 2000. Não houve delimitação por idioma. Estudos de revisão ou metanálise foram excluídos da busca. Somente artigos que comparavam via aberta e via endovascular no tratamento dos AAPs foram incluídos nesta revisão (incluindo manuscritos não randomizados e não controlados). 

 Assim, no dia 2 de setembro de 2017, foram acessados os portais de periódicos para realizar a pesquisa. Na PubMed, os descritores utilizados foram “popliteal aneurysm AND surgery AND endovascular” para artigos publicados nos últimos 20 anos, resultando em 300 artigos. Somente estudos que apresentavam resultados sobre a comparação entre as cirurgias aberta e endovascular no tratamento dos AAPs foram incluídos nesta revisão. Primeiramente, procedeu-se à leitura de título e resumo dos artigos e, caso houvesse dúvida quanto à presença ou não da comparação entre as técnicas, procedia-se à leitura do artigo completo. Desse modo, dos 300 artigos resultantes da busca, seis revisões foram excluídas e somente 14 artigos contemplaram os critérios de seleção utilizados. 

 Na LILACS, a busca foi realizada com os descritores “aneurisma AND poplítea AND cirurgia AND endovascular”, em português, espanhol e inglês, delimitando os resultados também para os últimos 20 anos. Dez artigos foram encontrados na busca em português, sendo os mesmos obtidos na busca em espanhol e inglês. Dois artigos eram revisão e foram excluídos da análise. Somente um artigo era comparativo e foi selecionado para esta revisão. 

 Quantos aos aspectos éticos, a pesquisa foi realizada exclusivamente em estudos publicados em bases de dados eletrônicas. Não foram realizadas pesquisas com seres humanos pelos pesquisadores e não foram utilizados dados confidenciais ou pessoais. Dessa forma, como foram utilizados dados de domínio público para a revisão bibliográfica, não houve necessidade de aprovação por parte do sistema CEP-CONEP. 

## RESULTADOS E DISCUSSÃO

 Foram analisados 15 artigos publicados entre os anos de 2005 e 2016 que compararam as técnicas de cirurgia aberta e cirurgia endovascular para o tratamento dos AAPs. Entre eles, 13 artigos eram estudos não randomizados 9 ^-^ 21 ; havia apenas um ensaio clínico randomizado 22 e um estudo prospectivo comparativo que deu continuidade à série estudada por esse ensaio clínico randomizado 23 . Nota-se, portanto, a dificuldade em se estabelecer um nível de evidência adequado para qualquer recomendação sobre a via de tratamento dos AAPs. 

 Com relação ao ano de publicação, um artigo foi publicado em 2005 22 , dois em 2007 19 ^,^ 23 , cinco 13 ^,^ 15 ^-^ 17 ^,^ 21 entre 2012 e 2014, e sete 9 ^-^ 12 ^,^ 14 ^,^ 18 ^,^ 20 entre 2015 e 2016. Todos foram publicados em revistas médicas, sendo o Journal of Vascular Surgery o periódico com mais publicações analisadas, sete 11 ^-^ 13 ^,^ 18 ^,^ 19 ^,^ 21 ^,^ 22 no total, seguido do European Journal of Vascular and Endovascular Surgery 14 ^,^ 16 e do Annals of Vascular Surgery, com duas 10 ^,^ 15 publicações cada. Houve um predomínio de publicações de estudos realizados nos Estados Unidos, com sete 10 ^,^ 11 ^,^ 13 ^,^ 17 ^-^ 19 ^,^ 21 artigos, seguidos da Itália com cinco 15 ^,^ 16 ^,^ 20 ^,^ 22 ^,^ 23 e Suécia 14 , Espanha 12 e Brasil 9 com um artigo cada. 

 Todos os artigos tinham como objetivo comparar as técnicas de cirurgia aberta e cirurgia endovascular utilizadas no tratamento dos AAPs. Os principais critérios analisados pelos artigos, em diferentes combinações, foram os seguintes: patência primária, patência secundária, taxa de reintervenção, mortalidade e amputação de membro. Um estudo também teve como objetivo definir a anatomia adequada da região para a não reincidência do aneurisma, além de definir os critérios ideais de seleção para o tratamento endovascular 10 . Outro estudo avaliou os resultados obtidos comparando as duas técnicas apenas no reparo de AAPs assintomáticos 11 . Modalidades distintas de reparo por via aberta (*bypass* venoso *versus bypass* com prótese) também foram comparadas com a via endovascular em um manuscrito 12 . 

 Um total de 5.166 procedimentos cirúrgicos de correção de AAPs foram comparados nos 15 artigos, sendo 3.930 cirurgias abertas e 1.236 cirurgias endovasculares. Onze estudos pesquisaram dados de um único centro de saúde, um utilizou dados de sete centros e três pesquisaram informações em grandes plataformas de dados de saúde, sendo elas Centers for Medicare & Medicaid Services, Vascular Quality Initiative e Swedvasc. 

 As comparações das duas modalidades cirúrgicas para o tratamento dos AAPs quanto à perviedade primária e/ou secundária nos diversos estudos foram realizadas em diferentes períodos de acompanhamento, desde 30 dias até 4 anos. Desse modo, devido à grande diversidade dos estudos e de seus resultados, foi difícil realizar uma comparação *head-to-head* entre as técnicas cirúrgicas. Análises estatísticas presentes em metanálises, em geral, selecionam apenas estudos randomizados e com rigorosa metodologia, algo não aplicável aos trabalhos presentes nesta revisão. 

 Com relação aos resultados encontrados nos estudos, observou-se uma variedade de situações quanto aos resultados das comparações entre a cirurgia aberta e a endovascular, dependendo da variável comparada e do tempo de acompanhamento. De modo geral, a cirurgia aberta, principalmente por *bypass* venoso, continua sendo o padrão-ouro para tratamento dos AAPs, especialmente em situações de emergência. Há, contudo, controvérsia na literatura sobre os resultados nessas situações. A via endovascular está mais indicada nas cirurgias eletivas, em pacientes de alto risco cirúrgico, resultando em menor tempo de permanência hospitalar e menos complicações precoces 9 ^,^ 13 ^,^ 14 . 

 Um estudo retrospectivo realizado na Itália comparou 43 casos de AAP submetidos a cirurgia aberta (grupo 1) com 21 casos submetidos a cirurgia endovascular (grupo 2). A avaliação de mortalidade e amputação após 30 dias do procedimento, patência primária, patência secundária e ausência de reintervenção aos 24 meses e ausência de amputação não mostrou diferenças significativas na comparação das técnicas aberta e endovascular. Entretanto, houve tendência a pior patência primária e maiores taxas de reintervenção no grupo endovascular. O tamanho da amostra e a alta porcentagem de pacientes sintomáticos abordados (48% no grupo 1 e 29% no grupo 2, demonstrando diferença na apresentação clínica entre os grupos) podem explicar parcialmente a falta de significância estatística. Além disso, os autores não apresentaram os dados de pacientes com quadro de isquemia aguda (32% no grupo 1 e 14% no grupo 2) separadamente, o que pode comprometer a comparação entre os dois procedimentos em reparo eletivo e emergencial 15 . 

 No ano seguinte, outro estudo retrospectivo realizado na Itália teve um maior tamanho amostral, de 174 pacientes submetidos a cirurgia aberta e 134 a cirurgia endovascular. Quando comparadas quanto a patência primária e secundária, ausência de reintervenção e preservação de membro, as duas técnicas apresentaram taxas absolutas semelhantes. As duas técnicas não foram comparadas entre si com análise estatística, somente análise descritiva. Os autores usaram como justificativa para esse fato as diferenças clínicas e anatômicas dos pacientes, mas acabaram realizando uma comparação direta entre as técnicas não confiável. Especificamente na via aberta, a patência primária aos 4 anos foi significativamente maior no *bypass* venoso comparado ao *bypass* com prótese. De maneira geral, a técnica aberta foi preferida em situações sintomáticas, com anatomia complexa e na presença de isquemia aguda que ameaçasse o membro. Por outro lado, houve reparo endovascular em situações emergenciais (7,5% na cirurgia endovascular e 17% na cirurgia aberta), sem apresentação específica desses resultados para comparação com a cirurgia eletiva 16 . 

 Pesquisa com dados do Medicare e Medicaid nos Estados Unidos totalizou 2.962 pacientes tratados com AAP (2.413 por via aberta e 549 por via endovascular). No grupo tratado por cirurgia endovascular, o número de reintervenções aos 30 e 90 dias de acompanhamento foram maiores que a cirurgia aberta, provavelmente devido à trombose de enxerto. Complicações, amputações de extremidade de membros e mortalidade aos 30 e 90 dias de acompanhamento não apresentaram diferenças significativas quando comparados os grupos. As complicações, embora semelhantes nos dois grupos, apresentaram variações. Por exemplo, o hematoma pós-operatório foi o mais comum na cirurgia endovascular, enquanto complicações cardiorrespiratórias e infecções foram mais frequentes na cirurgia aberta. O número de dias de internamento e os custos foram maiores no grupo tratado por cirurgia aberta. O grupo submetido a cirurgia endovascular apresentou mais pacientes com idade acima de 85 anos, o que pode ter contribuído para o maior número de complicações. Apesar disso, os autores concluíram que a cirurgia endovascular não tem benefícios em mortalidade, amputação e taxas de readmissão hospitalar quando comparada à cirurgia aberta 17 . 

 Outro estudo realizado nos Estados Unidos avaliou 35 pacientes submetidos a correção endovascular e 91 a cirurgia aberta. Ao se comparar as duas vias, aos 30 dias, as taxas de mortalidade, amputação, patência, complicações e reintervenção (chamados de eventos adversos maiores pelos autores) foram equivalentes entre as técnicas, independentemente da natureza emergencial ou eletiva. Entretanto, quando comparados os procedimentos eletivos e os emergenciais, as taxas de eventos adversos foram significativamente maiores nos pacientes de emergências, independentemente da via utilizada. Nas intervenções eletivas, a estimativa de 3 anos livre de reintervenção foi menor na via endovascular do que na aberta, bem como a tendência de superioridade nas taxas de eventos adversos maiores. Nas intervenções de emergências, após um ano, as taxas de eventos adversos maiores foram semelhantes em ambas as técnicas. Em resumo, ambas as vias são semelhantes quando o procedimento é realizado em casos de emergência, mas em procedimentos eletivos a via aberta apresenta vantagem em relação à endovascular, em um tempo maior de acompanhamento. As dificuldades encontradas no estudo foram pequeno número amostral, maior porcentagem de idosos com comorbidade no grupo endovascular e tempo de acompanhamento distinto entre as técnicas (2,6 anos na via endovascular e 3,8 anos na via aberta) 13 . 

 Um grupo de pesquisadores brasileiros comparou os resultados de 10 cirurgias endovasculares e 21 cirurgias abertas. A perviedade primária em 1 ano no grupo de via endovascular foi de 80%, enquanto no grupo de via aberta foi de 75%. Não houve diferença estatística aos 30 e 90 dias entre as técnicas com relação à sobrevida do membro. As complicações clínicas e cirúrgicas foram mais prevalentes na via aberta (19% e 10%, respectivamente). É importante ressaltar que 52,3% dos pacientes submetidos a via aberta apresentaram oclusão arterial aguda, uma emergência médica. Nenhum paciente submetido a via endovascular detinha quadro emergencial, sendo que 60% deles eram assintomáticos 9 . Como abordado em estudo já mencionado 13 , a cirurgia emergencial apresenta piores resultados quando comparada à eletiva. No estudo realizado no Brasil 9 , os autores usaram como justificativa para a não comparação entre as técnicas a heterogeneidade dos grupos (pacientes de alto risco cirúrgico para a via aberta, várias comorbidades e número de artérias de escoamento no grupo submetido a via endovascular e os casos de emergência submetidos a via aberta). Portanto, ao se analisar somente os resultados absolutos, o tratamento endovascular para AAP apresenta bons resultados em termos de perviedade, com taxas de complicações aceitáveis em pacientes com risco cirúrgico elevado e anatomia favorável. 

 Em avaliação de 171 AAPs tratados em 142 pacientes na Espanha, 139 aneurismas foram tratados por via aberta e 32 por via endovascular. O *bypass* venoso foi utilizado como padrão-ouro no tratamento de AAP, seguido de *bypass* com prótese e endoprótese. A patência primária após 30 dias foi semelhante nas três técnicas cirúrgicas. A patência primária e secundária, após 24 meses, foi significativamente maior no *bypass* venoso, sendo semelhante entre *bypass* com prótese e endovascular. Nesse estudo, nenhuma cirurgia de emergência foi realizada por via endoluminal, somente por via aberta (23%) 12 . Outro trabalho realizado nos Estados Unidos, com 30 pacientes submetidos a cirurgia por via aberta e 13 por via endovascular, demonstrou não haver diferença significativa entre as técnicas quanto às patências primária e secundária e mortalidade aos 24 meses. Entre as limitações desse estudo estão a pequena amostra e a presença de cinco pacientes com quadro emergencial tratados somente por via aberta, o que pode ter subestimado os resultados, sendo comparáveis à via endovascular 19 . 

 Em estudo sobre o tratamento exclusivo de AAPs assintomáticos realizado nos Estados Unidos, foram avaliados 390 pacientes, sendo 221 alocados ao grupo tratado por cirurgia aberta e 169 ao grupo tratado por cirurgia endovascular. O tempo de internação hospitalar foi significativamente maior na cirurgia aberta, porém com menores taxas de eventos adversos maiores (amputação ou reintervenção) após 1 ano 11 . Ronchey et al. 20 , por outro lado, avaliando na Itália 25 pacientes submetidos a cirurgia endovascular, 28 a cirurgia aberta por *bypass* venoso e 14 a cirurgia aberta por *bypass* protético, encontraram menor tempo de internação e necessidade de transfusão nos pacientes submetidos a cirurgia endovascular. Além disso, não houve diferença significativa entre os três grupos quanto à patência primária e secundária estimada em 5 anos e a reintervenções. Outro estudo nos Estados Unidos que comparou pacientes com 24 AAPs tratados com cirurgia endovascular e 63 com cirurgia aberta demonstrou resultados semelhantes, com tempo de hospitalização significativamente menor na via endovascular 21 . 

 Pacientes submetidos a cirurgia eletiva (n = 405) foram comparados a pacientes submetidos a cirurgia de emergência (n = 187) na Suécia para tratamento de AAP. Dos pacientes submetidos a cirurgia de emergência, 138 foram tratados por via aberta e 27 por via endovascular. Quando comparadas as técnicas, as patências primária e secundária, tanto após 30 dias quanto após 1 ano, foram maiores por via aberta (sendo maior no *bypass* venoso que no *bypass* com prótese) 14 . Esses resultados diferem do estudo que demonstrou resultados semelhantes entre as técnicas na apresentação emergencial 13 . No grupo eletivo sintomático, 90 AAPs foram tratados por via aberta e 13 por via endovascular. Nenhuma das variáveis comparadas entre os dois grupos foi significativa, entre elas: patência primária e secundária, amputação e óbito e amputação dentro de 30 dias e 1 ano. Já no grupo eletivo assintomático, 55 AAPs foram tratados por via endovascular e 245 por via aberta. Somente a patência primária dentro de 1 ano foi estatisticamente significativa a favor da cirurgia aberta (sendo maior no *bypass* venoso novamente) 14 . 

 Outro trabalho nos Estados Unidos avaliou 186 AAPs em 156 pacientes, dos quais 96 foram tratados por via aberta e 60 por via endovascular. A via aberta foi mais utilizada em pacientes com isquemia aguda, dor em repouso e trombose. Complicações dentro de 30 dias após a cirurgia e tempo de internação hospitalar foram significativamente maiores na via aberta, sendo que não houve diferenças na mortalidade após 30 dias ou amputação em relação à via endovascular. Patências primária e secundária aos 3 anos foram similares entre as duas técnicas. Não foi apresentada análise comparativa dos resultados das técnicas nos pacientes em quadro emergencial. Comparando exclusivamente os 130 pacientes eletivos (63 tratados com cirurgia por via aberta e 67 por via endovascular), sem trombose ou isquemia, a via aberta apresentou melhor patência primária aos 3 anos, mas sem diferença significativa em desfechos importantes como perda do membro ou patência secundária, devendo ser uma opção em pacientes saudáveis com boa expectativa de vida. Por outro lado, a via endovascular apresentou vantagem nos primeiros 30 dias pós-cirurgia, possibilitando menores complicações e alta hospitalar precoce 18 . 

 Tentando estabelecer critérios para a seleção de pacientes elegíveis para o procedimento endovascular no tratamento de AAPs, 77 procedimentos foram realizados em 66 pacientes também nos Estados Unidos. Desses, 52 foram tratados por via aberta e 25 por via endovascular. O tratamento endovascular foi indicado aos pacientes com alto risco cirúrgico, tortuosidade do vaso limitada, ausência de doença oclusiva significante (índice tornozelo-braço maior que 0,9) e AAP não envolvendo os segmentos abaixo do joelho. Os pacientes tratados por via endovascular eram mais velhos, com menor tempo de internação hospitalar e menores taxas de complicações. Tanto a patência primária como a patência secundária foram de 67,2% após 4 anos do reparo endovascular, sendo de 65,5% e 78,4%, respectivamente, para o reparo aberto. As técnicas cirúrgicas, assim como em outros estudos, não foram comparadas entre si, sendo os resultados apresentados apenas de forma descritiva 10 . 

 O ensaio clínico randomizado prospectivo 22 selecionado nesta revisão foi desenvolvido entre janeiro de 1999 e dezembro de 2003 na Itália, passando a ser um estudo comparativo prospectivo de janeiro de 2004 a dezembro de 2006 23 . No primeiro artigo, publicado em 2005, foram randomizados 15 pacientes em cada grupo, sendo que os tempos de cirurgia e de internação foram significativamente menores no grupo endovascular. As patências primária e secundária aos 4 anos de acompanhamento foram semelhantes entre os grupos 22 . 

 Dando continuidade à série, porém de maneira prospectiva comparativa e não randomizada, o trabalho publicado em 2007 comparou os resultados dos tratamentos de 27 AAPs por via aberta e 21 por via endovascular, apenas em pacientes assintomáticos. A patência primária foi de 100% na via aberta e 80,9% na via endovascular aos 12 meses, e de 71,4% e 88,1% aos 72 meses, respectivamente. A patência secundária aos 72 meses foi de 88,15% e 85,9% para as vias aberta e endovascular, respectivamente. Na comparação entre os grupos pelo teste de *log-rank,* não houve diferenças significativas. É importante ressaltar que este estudo teve um pequeno número amostral e que pacientes com menos de 50 anos de idade foram excluídos 23 . 

 Sobre o pequeno número amostral em estudos sobre o tema, o ensaio clínico randomizado *Open Versus Endovascular Repair of Popliteal Artery Aneurysm Trial* (OVERPAR), iniciado em 2013, foi cancelado devido a dificuldades no recrutamento de participantes 24 . 

## CONCLUSÕES

 Há ainda muita controvérsia na literatura acerca dos resultados da via endovascular no tratamento dos AAPs, sendo que o reparo aberto com *bypass* venoso continua sendo o padrão-ouro no tratamento dessa doença. O reparo endovascular apresenta um menor tempo de internação e é uma opção viável em pacientes eletivos, com baixa expectativa de vida, alto risco cirúrgico e presença de comorbidades e mais idosos, desde que com uma anatomia favorável para tal procedimento. O nível de evidência baseado nos estudos apresentados por esta revisão é B, fundamentado em um único estudo randomizado e diversos estudos não randomizados e não controlados. Assim, o bom senso e a experiência da equipe com as técnicas são importantes na decisão da indicação de determinada via para o tratamento dos AAPs. 

 Algumas ressalvas devem ser consideradas com relação aos resultados dos estudos que compararam as técnicas aberta e endovascular, pois a grande maioria dos estudos não era randomizada, incorrendo na possibilidade de viés de seleção. Foram observadas nesta revisão grandes diferenças nos tamanhos amostrais dos estudos, nos delineamentos metodológicos e nos tempos de acompanhamento pós-operatório. São necessários mais estudos comparativos de longo prazo para estabelecer os reais benefícios e indicações das técnicas a serem utilizadas, como o ensaio clínico randomizado controlado. 
