# Supplementary material for: Comparação entre cirurgia aberta e endovascular no tratamento do aneurisma da artéria poplítea: uma revisão
Source: J Vasc Bras. 2018 Jan-Mar;17(1):42–8. [Article in Portuguese] doi: 10.1590/1677-5449.008817 (PMC5990268; doi:10.1590/1677-5449.008817)
Supplement: Translation

# Translation: Comparison of open and endovascular surgery for treatment of popliteal artery aneurysm: a review

**Authors:** Ana Fernanda Fagundes Gonçalves, Carlos Augusto Pelek, Lorena Slusarz Nogueira, Renan Francisco de Carvalho, Matheo Augusto Morandi Stumpf, Ricardo Zanetti Gomes, Ana Claudia Garabeli Cavalli Kluthcovsky

**Affiliations:** 1 Universidade Estadual de Ponta Grossa – UEPG, Faculdade de Medicina, Ponta Grossa, PR, Brasil.

**Keywords:** aneurysm, popliteal artery, endovascular procedures

## Abstract

Popliteal artery aneurysms account for 70% of peripheral aneurysms and must be treated surgically. The results of endovascular treatment are controversial. The objective of this study is to conduct a literature review on comparisons between open surgery and endovascular treatment for popliteal artery aneurysms. Searches were run on the LILACS and MEDLINE databases using the appropriate search terms and 15 articles were selected. A total of 5,166 surgical procedures were compared, 3,930 open surgeries and 1,236 endovascular surgeries. Open surgery with venous bypass is still the gold standard. Endovascular surgery offers shorter length of hospital stay and is a viable option for elective patients, those with short life expectancy, high surgical risk, comorbidities, and more advanced age. However, long-term studies are needed to establish the true benefits and indications for the two techniques, such as randomized clinical trials.

## INTRODUCTION

 Popliteal artery aneurysms (PAAs) are the most common type of peripheral aneurysm, accounting for around 70% of cases. They are primarily found in the elderly and around 90% of patients are male. 1

 The deep anatomic position of the popliteal artery complicates clinical examination of the area, which in turn can make it less likely that PAAs will be diagnosed, particularly in asymptomatic cases. This type of aneurysm may be diagnosed because of acute thrombosis of the aneurysm (which presents as an acute arterial occlusion) or distal embolizations, or by calcifications visible on arteriography, palpation of a pulsating mass in the popliteal area during a physical examination, 1 or with imaging methods such as Doppler ultrasound, computed tomography angiography, or magnetic resonance angiography, and arteriography, in some cases. 1 ^-^ 3

 Surgery is recommended for popliteal aneurysms with diameters greater than two centimeters or those that are symptomatic. 4 Treatment may be with open surgery, which is the most widely-used approach, or endovascular surgery, using an endoprosthesis. 5

 Traditional open surgical treatment consists of proximal and distal ligature of the popliteal artery aneurysm to exclude the segment, with a venous bypass to divert flow around the aneurysm (although a prosthetic graft is also an option). The method can be simple if there is no need to expose the aneurysm, i.e., it is not necessary to section the tendons. However, there are also some disadvantages, since the proximal and distal ligation may miss some genicular branches, maintaining flow to the aneurysm and allowing it to grow even after surgery. This can cause compartment syndrome in neighboring structures, making additional surgery necessary. An alternative surgical technique is aneurysmectomy followed by interposition of a prosthetic bypass between the normal proximal and distal segments of the popliteal artery. However, the traditional approach is used more often. 1

 The endovascular technique is less invasive because an endograft is inserted via the groin, excluding the aneurysm sac from circulation. 6 For this procedure, the aneurysm must have proximal and distal necks with minimum lengths of 1 cm so that the endoprosthesis can be attached. However, because of its anatomical position, the popliteal artery has points of articulation to enable knee flexion. When the aneurysm extends beyond the knee joint, it is therefore necessary to use a flexible endoprosthesis that will fit the local anatomy. 7

 Although it is possible to treat PAAs either with open surgery or with endovascular techniques, 6 there is still controversy in the literature on the advantages of each technique in relation to the other. Additionally, few studies have been published in Brazil or internationally comparing the results of these two surgical approaches to treatment of PAAs. 

 The objective of this study was to conduct a literature review of studies that compare open surgery with endovascular treatment of PAAs. 

## MATERIAL AND METHODS

 This is a review of the literature comparing open surgery with endovascular surgery for treatment of PAAs. The material used to produce this article was sourced from the journal databases maintained by LILACS (Literatura Latino-Americana e do Caribe em Ciências da Saúde) and MEDLINE (Medical Literature Analysis and Retrieval System Online), via PubMed. 

 The PICO (patient, intervention, comparison, and outcomes) process was used to identify the essential elements of the research question and develop the strategy used to search for bibliographic evidence. 8 For this review, the descriptors “aneurysm AND popliteal” were used to define the patients to be studied; the descriptor used as intervention criterion was “surgery”; and the descriptor used for comparison to the intervention was “endovascular”. No descriptors were selected for outcome, because the articles analyzed were highly heterogeneous in terms of their objectives (some assessed primary patency during the first month and others years after treatment, while not all studies assessed length of hospital stay, for example). 

 The search period was set at the last 20 years, since use of endovascular treatment for PAA began during the 2000s. No restrictions were set on language of publication. Reviews and meta-analyses were excluded from the search. Only articles that compared the open and endovascular approaches for treatment of PAAs were included in the review (non-randomized and uncontrolled studies were included). 

 Thus, on September 2, 2017, the bibliographic databases were accessed and the searches were run. On PubMed, the terms used were “popliteal aneurysm AND surgery AND endovascular” for articles published in the last 20 years, and the search returned 300 articles. Only studies that reported results for the comparison between open surgery and endovascular treatment of PAAs were included in the review. The selection process began with reading of titles and abstracts and, in cases in which there was doubt as to whether the study included comparison between the techniques, the full text of the article was read. From the 300 articles initially identified, six review studies were excluded and just 14 articles were found that met the selection criteria. 

 On the LILACS database, searches were run using the terms “aneurysm AND popliteal AND surgery AND endovascular”, in Portuguese, Spanish, and English, and restricting results to the last 20 years. Ten articles were found searching in Portuguese, and the same articles were returned by the searches in Spanish and English. Two review articles were excluded and just one article contained comparison and was selected for the review. 

 With regard to ethical considerations, this study was conducted exclusively using published studies available on electronic databases. No research with human beings was conducted by the investigators and no confidential or personal data were used. As such, since data in the public domain were used for a bibliographic review, there was no need for approval from the national research ethics system. 

## RESULTS AND DISCUSSION

 Fifteen articles were analyzed, published between 2005 and 2016 and comparing open surgery and endovascular surgery for treatment of PAAs. Thirteen of these articles were non-randomized studies 9 ^-^ 21 ; there was just one randomized clinical trial 22 and one comparative prospective study which followed up on the series studied by this randomized clinical trial. 23 The difficulty of establishing an adequate evidence level for any recommendation on treatment approach for PAAs is therefore clear. 

 With relation to year of publication, one article was published in 2005, 22 two in 2007, 19 ^,^ 23 five 13 ^,^ 15 ^-^ 17 ^,^ 21 from 2012 to 2014, and seven 9 ^-^ 12 ^,^ 14 ^,^ 18 ^,^ 20 from 2015 to 2016. All were published in medical journals, and the Journal of Vascular Surgery was the periodical with the largest number of studies reviewed, at a total of seven, 11 ^-^ 13 ^,^ 18 ^,^ 19 ^,^ 21 ^,^ 22 followed by the European Journal of Vascular and Endovascular Surgery 14 ^,^ 16 and the Annals of Vascular Surgery, with two 10 ^,^ 15 publications each. The most common location of studies was the United States, with seven articles, 10 ^,^ 11 ^,^ 13 ^,^ 17 ^-^ 19 ^,^ 21 followed by Italy with five, 15 ^,^ 16 ^,^ 20 ^,^ 22 ^,^ 23 and then Sweden, 14 Spain, 12 and Brazil 9 with one article each. 

 All of the articles included the objective of comparing open surgery and endovascular techniques for treatment of PAAs. The principal criteria analyzed by the articles, in varying combinations, were as follows: primary patency, secondary patency, and rates of reintervention, mortality, and limb amputation. One study also had an objective of defining the most appropriate anatomy for the area to avoid recurrence of the aneurysm and to define the ideal selection criteria for endovascular treatment. 10 Another study evaluated the results obtained, comparing the two techniques for repair of asymptomatic PAAs only. 11 Different open repair modalities (venous bypass vs. bypass with prosthetic graft) were also compared with the endovascular approach in one article. 12

 A total of 5,166 surgical procedures to repair PAAs were compared in the 15 articles, breaking down as 3,930 open procedures and 1,236 endovascular procedures. Eleven studies analyzed data from a single health center, one used data from seven centers and three collected information from large health data platforms, specifically, the Centers for Medicare & Medicaid Services, the Vascular Quality Initiative, and Swedvasc. 

 Comparisons of primary and/or secondary patency after treatment of PAAs with the two different surgical methods were conducted at different points in time in different studies, ranging from 30 days to 4 years. Therefore, because of the great diversity in the studies and their results, it is difficult to make a “head-to-head” comparison between the two surgical techniques. In general, statistical analyses conducted in meta-analyses only select randomized studies and those with rigorous methodology, which is not the case of the studies reviewed here. 

 With relation to the results observed in the studies, there was a range of situations in terms of results of comparisons of open and endovascular surgery, depending on the variable compared and the follow-up period. In general, open surgery, and particularly venous bypass surgery, remains the gold standard for treatment of PAAs, especially in emergency situations. However, there is still controversy in the literature with relation to the results in these situations. The endovascular approach is preferred in elective surgeries and in patients with high surgical risk, and is associated with shorter length of hospital stay and fewer early complications. 9 ^,^ 13 ^,^ 14

 A retrospective study conducted in Italy compared 43 cases of PAA treated with open surgery (group 1) with 21 cases treated with endovascular surgery (group 2). Analyses of mortality and amputation 30 days after the procedure, primary patency, secondary patency, and freedom from reintervention at 24 months, and amputation-free limb survival did not reveal significant differences when open and endovascular techniques were compared. However, there were trends for poorer primary patency and higher reintervention rates in the endovascular group. The sample size and the high percentage of symptomatic patients (48% in group 1 and 29% in group 2, constituting a difference in clinical presentation between groups) may partially explain the lack of statistical significance. Additionally, the authors did not report data on patients with acute ischemia separately (32% in group 1 and 14% in group 2), which hampers comparison between the two procedures for elective and emergency repairs. 15

 Another retrospective study conducted in Italy the following year had a larger sample size, with 174 open surgery patients and 134 endovascular surgery patients. The two techniques had similar absolute rates for primary and secondary patency, freedom from reintervention, and limb salvage. They two techniques were not compared using statistical analysis, only with descriptive analysis. The authors explained this decision on the basis of the clinical and anatomic differences between patients, but the result was an unreliable direct comparison between the techniques. Specifically with relation to open surgery, 4-year primary patency was significantly higher after venous bypass than after prosthetic bypass. In general, the open technique was preferred in symptomatic cases, with complex anatomy, and when there was limb-threatening acute ischemia. However, although there were endovascular repairs in emergency situations (7.5% of endovascular surgeries and 17% of open surgeries), there was no specific presentation of these results to enable comparison with elective surgery. 16

 A study using data from Medicare and Medicaid in the United States analyzed 2,962 patients treated for PAA (2,413 with open and 549 with endovascular techniques). The numbers of reinterventions at 30 and 90 day follow-up were greater in the group treated with endovascular surgery, probably because of graft thrombosis. There were no significant differences between groups for complications, amputations of limb extremities, or mortality at 30 or 90-day follow-up. Although complication rates were similar in both groups, there were variations. For example, postoperative hematoma was most common with endovascular surgery, whereas cardiorespiratory complications and infections were more frequent with open surgery. The number of days in hospital was greater and costs were higher in the group treated with open surgery. The endovascular surgery group included more patients over the age of 85, which could have contributed to the greater number of complications. Additionally, the authors concluded that endovascular surgery does not offer benefits in terms of mortality, amputation, or hospital readmission rates when compared to open surgery. 17

 Another study conducted in the United States analyzed 35 patients who underwent endovascular repair and 91 treated with open surgery. Comparisons between the two approaches showed that at 30 days mortality rates, amputations, patency, complications, and reinterventions (referred to as major adverse events by the authors) were equivalent, irrespective of whether interventions were emergency or elective. However, when elective and emergency procedures were compared, rates of adverse events were significantly higher among emergency patients, irrespective of the approach employed. Among elective interventions, the estimated 3-year reintervention-free rate was lower for endovascular than for open surgery, and there was a trend for superiority in the rate of major adverse events. Among the emergency interventions, after 1 year rates of major adverse events were similar for both techniques. In summary, the methods are similar when the procedure is performed in emergency cases, but for elective procedures open surgery exhibited advantages over endovascular treatment over longer follow-up times. The limitations found in this study were a small sample, a higher percentage of elderly patients with comorbidities in the endovascular group and different follow-up periods for each method (2.6 years for endovascular and 3.8 years for open). 13

 A group of Brazilian researchers compared the results of 10 endovascular procedures and 21 open procedures. Primary patency at 1 year was 80% in the endovascular group and 75% in the open surgery group. There were no statistical differences between the two techniques for limb survival at 30 or 90 days. Clinical and surgical complications were more prevalent in the open group (19% and 10%, respectively). It is important to point out that 52.3% of the patients treated with open surgery had acute arterial occlusion, a medical emergency. None of the patients treated with endovascular repair was an emergency case, and 60% of them were asymptomatic. 9 As shown by a study already described above, 13 emergency surgery has worse results than elective. In the study conducted in Brazil, 9 the authors justified not comparing the techniques by the heterogeneous nature of the groups (patients with high surgical risk in the open group, several comorbidities, and number of runoff arteries in the endovascular group, and the emergency cases in the open group). As a result, analyzing just the absolute results, endovascular treatment for PAA had good patency rates and rates of complications were acceptable in patients with elevated surgical risk elevated and favorable anatomy. 

 In an analysis of 171 PAAs in 142 patients treated in Spain, 139 aneurysms were treated with open surgery and 32 via an endovascular approach. Venous bypass was used as the gold standard for PAA treatment, followed by prosthetic bypass and endoprosthesis. Primary patency at 30 days was similar for all three surgical techniques. Primary and secondary patency at 24 months were significantly higher for venous bypass and were similar for prosthetic bypass and endoprosthesis. This study did not include any emergency surgeries via the endoluminal route, all emergency surgery was open (23%). 12 Another study conducted in the United States, with 30 patients treated with open surgery and 13 with endovascular techniques, demonstrated no significant difference between the two methods in terms of primary or secondary patency or mortality at 24 months. This study’s limitations included a small sample and five emergency patients who were all treated with open surgery, which could have resulted in underestimation of the results of open surgery, which were similar to those of endovascular treatment. 19

 A study conducted in the United States exclusively analyzing treatment of asymptomatic PAAs investigated 390 patients, 221 in a group treated with open surgery and 169 in an endovascular surgery group. Length of hospital stay was significantly longer in the open surgery group, but the rates of major adverse events (amputation or reintervention) were lower at 1 year. 11 In Italy, Ronchey et al. 20 evaluated 25 patients treated with endovascular surgery, 28 with open surgery and venous bypass, and 14 with open surgery and prosthetic bypass, reporting a shorter length of hospital stay and reduced need for transfusion among patients treated with endovascular surgery. Additionally, there were no significant differences between the three groups in terms of primary or secondary patency estimated at 5 years or in terms of reinterventions. Another study in the United States compared patients with 24 PAAs treated with endovascular surgery and 63 with open surgery, demonstrating similar results, with length of hospital stay significantly shorter in the endovascular group. 21

 In Sweden, patients who underwent elective PAA surgery (n = 405) were compared with patients treated with emergency PAA surgery (n = 187). Among the emergency patients, 138 were treated with open surgery and 27 with endovascular techniques. When the techniques were compared, primary and secondary patency were better with open surgery at 30 days and at 1 year (and were higher for venous bypass than for bypass with prosthetic graft). 14 These results differ from those of a study that demonstrated similar results for the two techniques in emergency cases. 13 In a symptomatic elective group, 90 PAAs were repaired with open surgery and 13 with endovascular treatment. None of the variables used to compare the two groups were significant. These included: primary and secondary patency, amputation, and death and amputation at 30 days and 1 year. In the elective asymptomatic group, 55 PAAs were treated with endovascular techniques and 245 by open surgery. Only primary patency within 1 year was statistically significant in favor of open surgery (more so for venous bypass, once more). 14

 Another study conducted in the United States analyzed 186 PAAs in 156 patients, 96 of which were treated with open surgery and 60 via an endovascular approach. Open surgery was more often used in patients with acute ischemia, pain at rest, and thrombosis. The rate of complications within 30 days after surgery, and length of hospital stay were significantly greater in the open group, and there were no differences in mortality at 30 days or amputation in comparison with the endovascular group. Primary and secondary patencies at 3 years were similar for both techniques. No comparative analysis was reported for the results of the two techniques in emergency patients. However, comparing only the 130 elective patients (63 treated with open surgery and 67 via an endovascular approach), without thrombosis or ischemia, the open group had better primary patency at 3 years, but there were no significant differences in important outcomes such as limb loss or secondary patency, and so open surgery is an option for healthy patients with good life expectancy. On the other hand, endovascular treatment offered advantages during the first 30 days after surgery, with fewer complications and earlier hospital discharge. 18

 In a study in the United States that attempted to establish criteria for selection of patients eligible for endovascular treatment of PAAs, 77 procedures were performed in 66 patients. Of these, 52 were treated with open surgery and 25 with endovascular techniques. Endovascular treatment was indicated for patients with high surgical risk, limited vessel tortuosity, absence of significant occlusive disease (ankle-brachial index greater than 0.9) and PAA that did not involve segments below the knee. Patients treated via an endovascular approach were older, with shorter length of hospital stay, and lower rates of complications. Both primary and secondary patency were 67.2% 4 years after endovascular repair, and 65.5% and 78.4%, respectively, in the open surgery group. As in other studies, the surgical techniques were not compared and the results were only reported in descriptive form. 10

 The prospective randomized clinical trial 22 selected for this review was conducted from January 1999 to December 2003 in Italy, and became a comparative prospective study from January 2004 to December 2006. 23 In the first article, published in 2005, 15 patients were randomized into each group and duration of surgery and hospital stay were significantly shorter in the endovascular group. Primary and secondary patencies at 4-year follow-up were similar in both groups. 22

 The 2007 study continued the series with a prospective, non-randomized, comparative design, comparing the results of open treatment of 27 PAAs with endovascular treatment of 21 PAAs, all in asymptomatic patients. Primary patency at 12 months was 100% after open surgery and 80.9% after endovascular treatment, and 71.4% and 88.1% at 72 months, respectively. Secondary patency at 72 months was 88.15% and 85.9% for open and endovascular patients, respectively. Comparison of the groups with the log-rank test indicated no significant differences. It is important to point out that this study had a small sample and patients under the age of 50 years were excluded. 23

 With regard to the small sample sizes in studies of this subject, the randomized clinical trial Open Vs. Endovascular Repair of Popliteal Artery Aneurysm Trial (OVERPAR), started in 2013, was cancelled because of difficulties recruiting participants. 24

## CONCLUSIONS

 There is still significant controversy in the literature with relation to the results of endovascular treatment of PAAs, and open repair with venous bypass remains the gold standard for treatment of this disease. Endovascular repair offers shorter length of hospital stay and is a viable option in elective patients, with short life expectancy, high surgical risk and comorbidities and among older patients, as long as they have anatomy that is favorable for the procedure. The evidence level supported by the studies analyzed in this review is B, founded on a single randomized study and several non-randomized and uncontrolled studies. Therefore, the treating team’s best judgment and experience with the techniques are important elements in decision-making on which method to use to treat PAAs. 

 Certain provisos should be noted with relation to the results of studies that compared the open and endovascular techniques, since the great majority were not randomized, allowing for the possibility of selection bias. In this review, there were major differences between studies in terms of sample sizes, methodological design, and postoperative follow-up periods. Further comparative and long-term studies are needed to establish the true benefits and indications of the techniques available, such as a randomized and controlled clinical trial.
